# *Hystrignathus dearmasi* sp. n. (Oxyurida, Hystrignathidae), first record of a nematode parasitizing a Panamanian Passalidae (Insecta, Coleoptera)

**DOI:** 10.3897/zookeys.57.477

**Published:** 2010-09-21

**Authors:** Jans Morffe, Nayla García

**Affiliations:** Instituto de Ecología y Sistemática, Carretera de Varona km 31/2, Capdevila, Boyeros, A.P. 8029, C.P. 10800, Ciudad de La Habana, Cuba

**Keywords:** Nematoda, Hystrignathidae, Hystrignathus, Passalidae, Passalus, Panama

## Abstract

Hystrignathus dearmasi sp. n. (Oxyurida: Hystrignathidae) is described from an unidentified passalid beetle (Coleoptera: Passalidae) from Panama. It resembles Hystrignathus cobbi Travassos & Kloss, 1957 from Brazil, by having a similar form of the cephalic end, extension of cervical spines and absence of lateral alae. It differs from the latter species by having the body shorter, the oesophagus and tail comparatively larger, the vulva situated more posterior and the eggs ridged. This species constitutes the first record of a nematode parasitizing a Panamanian passalid.

## Introduction

The family Hystrignathidae includes a large number of monoxenous nematodes from passalid beetles. At present, more than 100 species have been described from North America, Mexico, Cuba, Lesser Antilles, Brazil, Africa, Madagascar and Australasia.

The type genus of the family, Hystrignathus Leidy, 1850, is characterized by having a single cephalic annule, the cervical cuticle armed with opposite rows of spines, procorpus clavate and genital tract didelphic-amphidelphic (Adamson and Van Waerebeke 1992). At present, 21 species are described, 11 of which are from Brazil, where the group have received major attention (Travassos and Kloss 1957a, b, 1958). The rest of the nominal species are known from North America, Cuba, Trinidad, Venezuela, Ivory Coast and Madagascar (Leidy 1850, Van Waerebeke 1973, Guerrero 1980, Hunt 1982, Van Waerebeke and Remillet 1982, García et al. 2009).

The family Passalidae in Panama comprises about 60 species belonging to 16 genera (de Armas, pers. comm.). Despite such diversity there are no records of parasitic nematodes from Panamanian passalid beetles. In general, parasitological surveys of passalids are scarce in Central America. The few studies that have been carried out are restricted to the area of the Yucatan peninsula, Mexico (Coy and García 1995, García and Coy 1997).

In this paper a new species of Hystrignathus from Panama is described. It constitutes the first record of a parasitic nematode from passalid beetles for this country.

## Material and methods

Two specimens of an unidentified small, blackish passalid beetle were collected by hand on rotting logs from the Summit National Park, Panama Province, Panama.

Hosts were killed by decapitation and the last abdominal segments were removed in order to extract the guts that were fixed and conserved in 70% ethanol. Intestines were dissected as soon as possible in Petri dishes with 70% ethanol under a stereomicroscope. The nematodes found were removed and fixed in 70% ethanol.

Nematodes were transferred and cleared in glycerine via slow evaporation method and mounted in the same medium. The edges of the coverslips were sealed using nail polish. Measurements were taken as in Morffe et al. (2009) and are expressed in millimetres, except where indicated. De Man’s ratios a, b, c and V% were calculated. Each variable is shown as the range followed by the mean plus standard deviation in parentheses, the number of measurements is also given. Micrographs were obtained with the aid of an AxioCam digital camera attached to a Carl Zeiss AxioScop 2 Plus compound microscope. Line drawings were made with the softwares CorelDRAW X3 and Adobe Photoshop CS2 using the micrographs as templates. Scale bars of all plates are given in millimetres.

The type-material is deposited in the Colección Helmintológica de las Colecciones Zoológicas (CZACC) from the Instituto de Ecología y Sistemática, Havana, Cuba and the Coleçao Helmintologica do Instituto Oswaldo Cruz (CHIOC), Rio de Janeiro, Brazil.

## Systematics

Genus Hystrignathus Leidy, 1850

### Hystrignathus dearmasi sp. n.

urn:lsid:zoobank.org:act:62307E25-9A1E-4CE0-9571-B5AE5B7E733C

**Figure 1.**
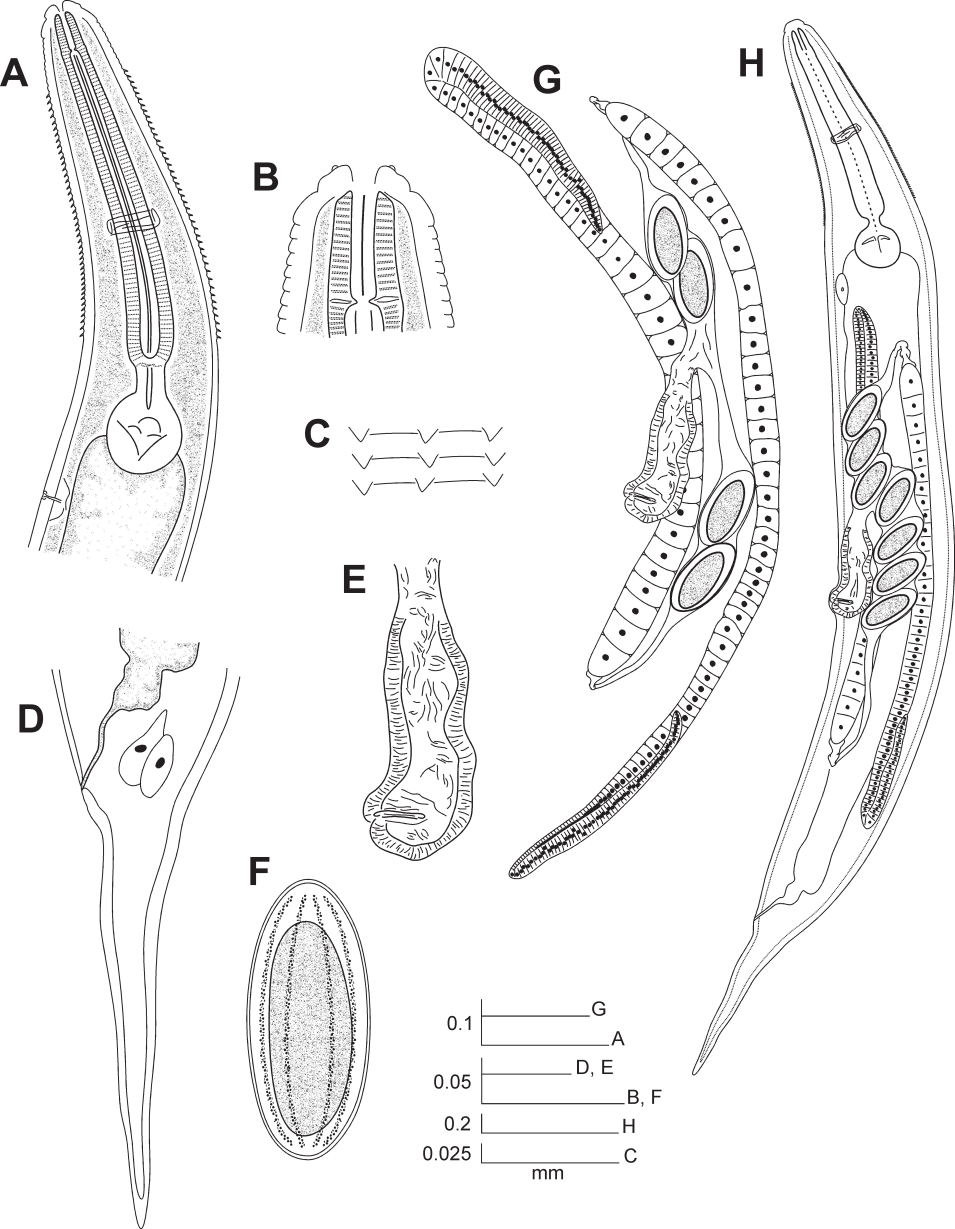
Hystrignathus dearmasi sp. n. female. **A** Esophageal region, lateral view **B** Cephalic region and stoma **C** Cervical spines **D** Tail, lateral view **E** Vulva, ventro-lateral view **F** Egg **G** Genital tracts **H** Entire nematode.

**Figure 2.**
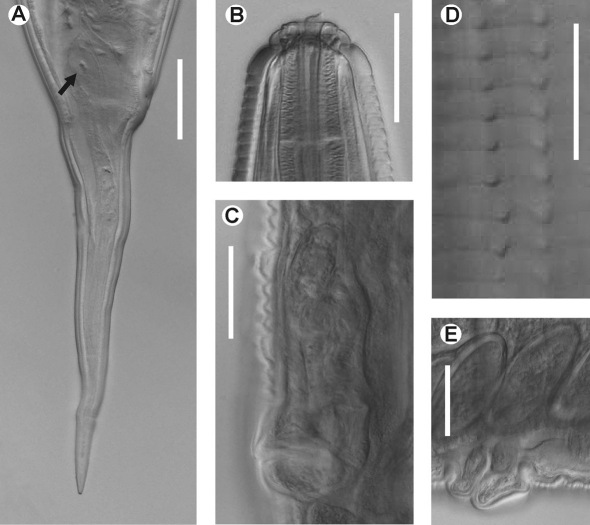
Hystrignathus dearmasi sp. n. female. **A** Tail, lateral view (arrow shows therectal glands) **B** Cephalic region and stoma **C** Vulva, ventro-lateral view **D** Cervical spines **E** Prominent lips of the vulva, lateral view. Scale bars: A, B, C, E. 0.05 mm; D. 0.025 mm.

Fig. 1 A–H Fig. 2 A–E 

#### Type material.

♀ holotype, Panama, Panama Province, Summit National Park; in unidentified short, blackish Passalidae; 25.IX.2009; L. F. de Armas coll.; CZACC 11.4604. Paratypes: 6 ♀♀, same data as holotype, CZACC 11.4605–11.4610; 2 ♀♀, CHIOC, same data as holotype.

#### Measurements.

Holotype (female) a = 8.62, b = 4.89, c = 7.24, V% = 57.46, total length = 1.810, maximum body width = 0.210, first cephalic annule (length×width) = 0.013×0.055, stoma length = 0.045, procorpus length = 0.295, isthmus length = 0.025, diameter of basal bulb = 0.090, total length of oesophagus = 0.370, nerve ring to anterior end = 0.213, excretory pore to anterior end = 0.480, vulva to posterior end = 0.770, anus to posterior end = 0.250, eggs = 0.095–0.110×0.043–0.048 (0.099 ± 0.007×0.046 ± 0.002 n = 4).

Paratypes (females) (n = 8): a = 7.81–9.37 (8.61 ± 0.58 n = 8), b = 4.17–5.35 (4.84 ± 0.51 n = 6), c = 6.19–8.16 (7.14 ± 0.59 n = 8), V% = 54.27–60.00 (56.69 ± 2.05 n = 7), total length = 1.300–1.780 (1.549 ± 0.179 n = 8), maximum body width = 0.158–0.210 (0.180 ± 0.019 n = 8), first cephalic annule (length×width) = 0.010–0.015×0.048–0.055 (0.013 ± 0.001×0.052 ± 0.003 n = 7), stoma length = 0.038–0.045 (0.041 ± 0.004 n = 8), procorpus length = 0.230–0.273 (0.248 ± 0.016 n = 8), isthmus length = 0.020 (n = 1), diameter of basal bulb = 0.070–0.085 (0.077 ± 0.005 n = 8), total length of oesophagus = 0.300–0.350 (0.325 ± 0.019 n = 6), nerve ring to anterior end = 0.175–0.190 (0.184 ± 0.007 n = 4), excretory pore to anterior end = 0.420–0.450 (0.433 ± 0.015 n = 4), vulva to posterior end = 0.590–0.770 (0.686 ± 0.073 n = 7), anus to posterior end = 0.190–0.250 (0.218 ± 0.023 n = 8), eggs = 0.088–0.103×0.038–0.055 (0.097 ± 0.005×0.046 ± 0.004 n = 16).

#### Description.

Female body robust, slightly fusiform. Cuticle strongly annulated in spiny region (annule c.5 µm width) and less in rest of body. Cervical cuticle armed with spines from some distance beyond stoma (distance about length of stoma) almost to end of procorpus. Spines arranged initially in c. 16 apposite rows that do not seem to increase consistently where they terminate. Anterior spines short and wide, scale-like, becoming sharply pointed but still short toward end of rows. Sub-cuticular longitudinal striae present. Lateral alae absent. Head bearing 8 paired papillae, set-off from body by single groove. First cephalic annule cone-like and truncated, not inflated, c.1.5 head lengths long. Stoma short, wide, about 4 first annule lengths long, surrounded by oesophageal collar. Oesophagus consists of muscular procorpus whose diameter increases slightly and gradually, well set-off from short isthmus. Intestine simple, sub-rectilinear, its fore region inflated. Rectum short, anus not prominent. At least with 2, large, ovoid, rectal glands with central nuclei at level of rectum. Nerve ring encircles procorpus at about its midpoint. Excretory pore located at about half of body width posterior to basal bulb. Vulva a median transverse slit slightly displaced towards posterior half of body, lips very prominent. Vagina muscular, forwardly directed. Genital tract didelphic-amphidelphic. Ovaries reflexed. Anterior ovary shorter, reflexed just posterior excretory pore, posterior ovary reflexed at slightly more than body width before anus. Both flexures about 2 body-widths long. Eggs ovoid, numerous, bearing 8 longitudinal, slightly prominent ridges on shell. Tail comparatively short, conical, attenuated, sharply pointed. Male unknown.

#### Differential diagnosis.

 Hystrignathus dearmasi sp. n. is similar to Hystrignathus cobbi Travassos & Kloss, 1957 from Brazil, since both have a similar form of the cephalic end, spines commencing posterior to the stoma (feature unique in the genus) and the apparent absence of lateral alae (Travassos and Kloss 1957b). From the latter, Hystrignathus dearmasi sp. n. differs by having the body consistently shorter (1.300–1.81 *vs.* 2.432–2.79), but the tail (c = 6.19–8.16 *vs.* 14.48–16.61) and oesophagus (b = 4.17–5.35 *vs.* 5.68–6.30) comparatively longer. The vulva is located further forward in Hystrignathus dearmasi sp. n. (V%: 54.27–60.00 *vs.* 62.25–64.37). The eggs of Hystrignathus cobbi have smooth shells instead of the ridged eggs of Hystrignathus dearmasi sp. n.

Hystrignathus heliae Travassos & Kloss, 1957, from Brazil, has a similar cephalic end, but can be differentiated by the length of the stoma, which hardly surpasses the base of the first cephalic annule, and spines starting at the end of the cephalic annule. In Hystrignathus dearmasi sp. n. the stoma is notably longer and the spines commence at some distance posterior to it.

#### Type host.

 Unidentified, short, blackish passalid beetle (Coleoptera: Passalidae).

#### Site.

 Gut caeca.

#### Type locality.

 Summit National Park, Panama Province, Panama.

#### Etymology.

 The specific epithet honours Dr. Luis F. de Armas Chaviano, an eminent Cuban aracnologist and the collector of the type-host.

### Key to the species of the genus Hystrignathus

Note: In the following key we omit two species of Cuban hystrignathids formerly placed in the genus Hystrignathus, because they will be published in the future as new combinations.

**Table:** 

1.	Rows of spines commencing to some distance posterior to the stoma	2
–	Rows of spines commencing just after the end of the first cephalic annule	3
2.	Tail very short (c = 14.48–16.61); eggs with smooth shell	Hystrignathus cobbi Travassos & Kloss, 1957
–	Tail longer (c = 6.19–8.16); eggs with less prominent ridges on the shell	Hystrignathus dearmasi Morffe & García sp. n.
3.	One ovary atrophied	Hystrignathus inegalis Van Waerebeke & Remillet, 1982
–	Both ovaries well developed	4
4.	First cephalic annule long and notably inflated	5
–	First cephalic annule shorter and less inflated	7
5.	Eggs with smooth shell	6
–	Eggs with ridged shell	Hystrignathus splendidus Morffe & García, 2010
6.	Oesophagus longer than the tail	Hystrignathus tarda (Artigas, 1928)
–	Oesophagus as longer as the tail	Hystrignathus inflatus Travassos & Kloss, 1957
7.	Stoma not extending further than end of the first cephalic annule	8
–	Stoma extending further than end of the first cephalic annule	9
8.	Spines ending at the level of the excretory pore; tail longer (c =8.63)	Hystrignathus paulistanus Cordeira, 1981
–	Spines ending at the end of the basal bulb; tail shorter (c = 9.61)	Hystrignathus papillophorus Cordeira, 1981
9.	Eggs with a ridged shell	10
–	Eggs with a smooth shell	15
10.	First cephalic annule very short, much less than half the stoma length	11
–	First cephalic annule longer, about half the stoma length	Hystrignathus metropolitanus Cordeira, 1981
11.	Lateral alae surpass the level of the vulva	12
–	Lateral alae do not surpass the level of the vulva	13
12.	Tail markedly attenuate and comparatively short (c = 6.0–7.6)	Hystrignathus egalis Van Waerebeke & Remillet, 1982
–	Tail markedly subulate and comparatively large (c = 3.64–4.81)	Hystrignathus rescens Travassos & Kloss, 1958
13.	Spines terminate at a short distance (less than a body-width) posterior to basal bulb	14
–	SSpines terminate at a longer distance (about a body-width) posterior to basal bulb	Hystrignathus ferox Hunt, 1982
14.	Lateral alae end at the level of the vulva; tail comparatively larger (c = 3.38–3.98)	Hystrignathus rosario García, Ventosa & Morffe, 2009
–	Lateral alae end before the level of the vulva; tail comparatively shorter (c = 5.71–6.86)	Hystrignathus rugosus Travassos & Kloss, 1958
15.	Spines terminate before the basal bulb	16
–	Spines terminate after the basal bulb	19
16.	Lateral alae present	17
–	Lateral alae not present	Hystrignathus popiliophagus Guerrero, 1980
17.	Spines cease at the end of the bulb; tail very short (c = 7.88–10.66)	Hystrignathus heliae Travassos & Kloss, 1957
–	Spines cease before the end of the bulb; tail longer (c ≤ 7)	18
18.	Lateral alae end just before the anus	Hystrignathus insularis Van Waerebeke, 1973
–	Lateral alae end at certain distance before the anus	Hystrignathus meridensis Guerrero, 1980
19.	Spines terminate at the level of the excretory pore	20
–	Spines terminate slightly anterior to the excretory pore	Hystrignathus rigidus Leidy, 1850
20.	Stoma very short, hardly surpassing the end of the first cephalic annule	Hystrignathus pearsoni Travassos & Kloss, 1958
–	Stoma longer, clearly surpassing the end of the first cephalic annule	Hystrignathus spinosus Travassos & Kloss, 1957

## Supplementary Material

XML Treatment for Hystrignathus dearmasi
